# Worldwide Incidence of Colorectal Cancer, Leukemia, and Lymphoma in Inflammatory Bowel Disease: An Updated Systematic Review and Meta-Analysis

**DOI:** 10.1155/2016/1632439

**Published:** 2016-05-16

**Authors:** Chelle L. Wheat, Kindra Clark-Snustad, Beth Devine, David Grembowski, Timothy A. Thornton, Cynthia W. Ko

**Affiliations:** ^1^School of Public Health, Department of Health Services, University of Washington, Seattle, WA 98195, USA; ^2^Division of Gastroenterology, Department of Medicine, University of Washington, Seattle, WA 98195, USA; ^3^School of Pharmacy, Department of Pharmacy, University of Washington, Seattle, WA 98195, USA; ^4^School of Public Health, Department of Biostatistics, University of Washington, Seattle, WA 98195, USA

## Abstract

*Background/Aims*. Inflammatory bowel disease (IBD) is associated with an increased risk of colorectal cancer (CRC). In addition, there may be an association between leukemia and lymphoma and IBD. We conducted a systematic review and meta-analysis of the IBD literature to estimate the incidence of CRC, leukemia, and lymphoma in adult IBD patients.* Methods*. Studies were identified by a literature search of PubMed, Cochrane Library, Medline, Web of Science, Scopus, EMBASE, and ProQuest Dissertations and Theses. Pooled incidence rates (per 100,000 person-years [py]) were calculated through use of a random effects model, unless substantial heterogeneity prevented pooling of estimates. Several stratified analyses and metaregression were performed to explore potential study heterogeneity and bias.* Results*. Thirty-six articles fulfilled the inclusion criteria. For CRC, the pooled incidence rate in CD was 53.3/100,000 py (95% CI 46.3–60.3/100,000). The incidence of leukemia was 1.5/100,000 py (95% CI −0.06–3.0/100,000) in IBD, 0.3/100,000 py (95% CI −1.0–1.6/100,000) in CD, and 13.0/100,000 py (95% CI 5.8–20.3/100,000) in UC. For lymphoma, the pooled incidence rate in CD was 0.8/100,000 py (95% CI −0.4–2.1/100,000). Substantial heterogeneity prevented the pooling of other incidence estimates.* Conclusion*. The incidence of CRC, leukemia, and lymphoma in IBD is low.

## 1. Introduction

Colorectal cancer (CRC) incidence is higher in inflammatory bowel disease (IBD) patients than in the general population, and CRC accounts for an estimated 10–15% of deaths in patients with IBD [1]. The risk conferred by IBD may be due to chronic inflammation combined with genetic factors [1–3]. Patients with extensive inflammation, a younger age at diagnosis, long disease duration, comorbid primary sclerosing cholangitis (PSC), and pseudopolyposis are at the highest risk [4–14].

IBD patients receiving immunomodulators may or may not also be at higher risk of lymphoproliferative disorders such as lymphoma and leukemia [15–19]. The risk of lymphoma in IBD patients is low but appears to be higher than in the general population [6, 8, 14, 20–22]. The risk of leukemia in IBD is less clear [6, 8, 14, 23, 24].

Understanding the risk of development of these malignancies inherent to IBD is crucial for cancer surveillance strategies. In addition, determination of the absolute increase in risk of these malignancies from IBD pharmacotherapy is a crucial consideration for providers and patients. The aims of this study are to estimate the incidence of CRC, leukemia, and lymphoma in adult IBD patients through a systematic review and meta-analysis. Unique to this study, we attempt to evaluate the underlying risk of these cancers in IBD overall and separately Crohn's Disease (CD) and ulcerative colitis (UC) and exclude the effects of IBD pharmacotherapy (specifically immunomodulators and biologics), given the evidence that these medications may increase cancer risk.

## 2. Materials and Methods

### 2.1. Literature Search

**Figure 1:**
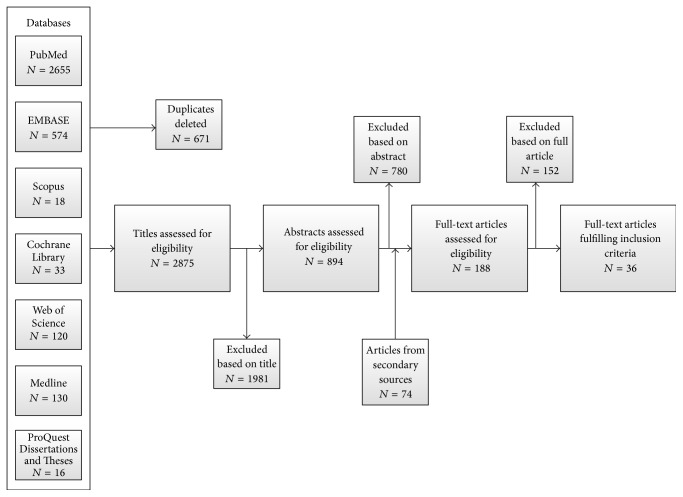
Flowchart depicting the identification of studies, inclusion, and exclusion assessment.

A detailed literature search was conducted to identify all published and unpublished studies examining the incidence of CRC, leukemia, and lymphoma in adult IBD patients. We searched the PubMed, Cochrane Library, Medline, Web of Science, Scopus, EMBASE, and ProQuest Dissertations and Theses databases. Reference lists of published articles were hand searched for secondary sources and experts in the field contacted for unpublished data. Furthermore, https://clinicaltrials.gov/, the WHO International Clinical Trial Registry, and scientific information packets of approved IBD pharmacotherapies were scrutinized for additional information sources. No restrictions on language, country of origin, or publication date were used.  Figure 1 outlines the literature search and Supplementary Table  1 (in Supplementary Material available online at http://dx.doi.org/10.1155/2016/1632439) details the search strategy employed.

### 2.2. Inclusion and Exclusion Criteria

All studies that reported incidence or provided information sufficient to accurately calculate incidence for the three cancers of interest in adult IBD patients were included. Studies focusing on pediatric populations, not reporting person-years of follow-up, of duration less than one year, and not written in English and unable to be translated to English were excluded. If publications reported duplicate data on a population, only the publication with the longest follow-up period was included.

### 2.3. Data Collection and Quality Assessment

Two independent reviewers (CW and KCS) examined each article for inclusion according to the eligibility criteria. Any disagreement was resolved through discussion and consensus. Thirty-six articles fulfilled the inclusion criteria. Twenty-five articles reported incidence estimates for CRC [7, 10, 11, 13, 14, 18, 19, 21, 22, 24–39], ten for leukemia [8, 14, 18, 19, 21–24, 33, 39], and twenty-one for lymphoma [8, 10, 14, 18, 19, 21, 22, 24, 26, 33, 34, 39–48] (some articles reported incidence estimates for multiple cancers).  Figure 1 outlines the search flowchart.

We retrieved demographic (where possible) and outcome data for each included article using standardized forms. Individual studies were assigned a bias risk rating using the Cochrane Collaboration's Risk of Bias Assessment Tool: for Non-Randomized Studies of Interventions (ACROBAT-NRSI) [49]. The strength of evidence for each cancer was assessed utilizing the Grades of Recommendation, Assessment, Development, and Evaluation (GRADE) approach [50].

### 2.4. Statistical Analysis

Individual study unadjusted incidence rates (per 100,000 person-years [py]) were calculated from the reported number of cancer cases and person-years of follow-up for each outcome separately. Standard errors and 95% confidence intervals (CIs) were estimated assuming a Poisson distribution [51]. In situations with zero observed cases, the value of 3.7 was used to calculate incidence rates and the confidence interval upper limit [51].

As our interest is in quantifying the incidence rate of CRC, leukemia, and lymphoma in IBD patients not treated with immunomodulators or biologic agents (and treatment information is often unreported), two stratification variables were created using study publication year as an estimate of when each medication class became widely used. 1995 was used as the dividing year for widespread immunomodulator use and 2000 for biologic use. Pooled incidence rates with 95% CIs were then calculated for (1) each cancer overall, (2) each cancer in CD and UC separately, (3) each cancer stratified by year of publication, and (4) each cancer stratified by country of origin (to determine if incidence varied by geographic region). A random effects model was used to account for potential between-study variations. The *I* ^2^ statistic was used to quantify the percentage of heterogeneity for all pooled estimates from between-study variation, with ≥75% indicating substantial heterogeneity [52]. Publication bias and the presence of other small study effects were measured through visual assessment of funnel plot symmetry and Egger's test [52]. Sensitivity analyses were undertaken to explore potential sources of heterogeneity. Metaregression was used to further test the effects of study- and subject-level covariates on cancer risk, as well as the degree of between-study heterogeneity explained by the covariates through calculation of the adjusted *R* ^2^. The adjusted *R* ^2^ measures the relative reduction in the between-study variance explained by the covariates in the model and is presented as a percentage [52]. Statistical analysis was performed using Stata (StataCorp, College Station, TX). *p* values ≤0.05 were considered statistically significant.

## 3. Results and Discussion

### 3.1. Results

#### 3.1.1. Colorectal Cancer

Reported incidence rates of CRC in IBD ranged from 41.5/100,000 py (95% CI 24.5–58.5/100,000) to 543.5/100,000 py (95% CI 316.4–770.6/100,000) (Table 1). Substantial heterogeneity prevented pooling of estimates using a random effects model (heterogeneity test, chi^2^ = 174.65; *p* < 0.001; *I* ^2^ = 86.3%). Therefore, we present unpooled incidence estimates. Separate sensitivity analyses excluding the studies with the highest individual incidence estimate [31] and the study with the greatest weight on the pooled estimate [7] did not significantly change the degree of heterogeneity present.

**Figure 2:**
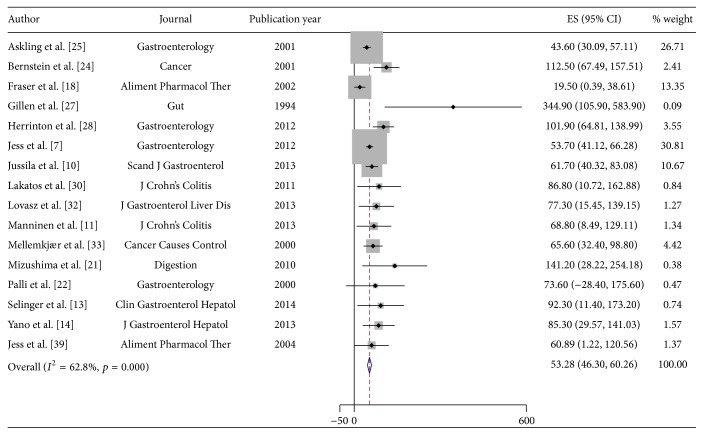
Incidence of colorectal cancer (CRC) in patients with Crohn's Disease (CD). Each incidence estimate is presented followed by the 95% confidence intervals (CIs). Each square in the plot indicates the point estimate of the incidence. The diamond represents the summary incidence from the pooled studies. Error bars depict the 95% CIs.

Reported CRC incidence rates in CD ranged from 19.5/100,000 py (95% CI 0.4–38.6/100,000) to 344.9/100,000 py (95% CI 105.9–583.9/100,000) (Table 1). Using a random effects model, an estimated incidence of CRC in CD of 53.3/100,000 py (95% CI 46.3–60.3/100,000) was obtained.  Figure 2 displays the Forest plot for the pooled estimates. In UC, the reported incidence rates ranged from 54.5/100,000 py (95% CI 30.0–79.0/100,000) to 543.5/100,000 py (95% CI 316.4–770.6/100,000). Substantial heterogeneity was again present when pooling using a random effects model (heterogeneity test, chi^2^ = 110.7; *p* < 0.001; *I* ^2^ = 86.4%), and thus the results in UC were not pooled.

Analyses stratified by publication year and region of origin did not reveal any significant differences in results. We also conducted metaregression analyses to evaluate the potential impact of age, gender, race, Montreal Classification, disease duration, surgical history, smoking status, comorbid primary sclerosing cholangitis, presence of extraintestinal manifestations, and concomitant treatment with immunosuppressants and/or biologics on the CRC incidence in IBD. Due to the limited sample size and incomplete reporting of demographic characteristics in many studies, these analyses were underpowered. Together, age, gender, and disease duration explained a significant proportion of the between-study variability (adjusted *R* ^2^ = 65.67%); however we could not make any further conclusions regarding the impact of these covariates on CRC incidence in IBD. Evaluation of funnel plots and Egger's test showed evidence of small study effects and/or publication bias for IBD overall (*p* = 0.149) and weak evidence of small study effects in CD and UC (*p* = 0.005 CD; *p* = 0.05 UC). However, the power of these tests may be compromised due to small sample sizes and significant heterogeneity between studies. Given the observational nature of the included studies and the probability of bias from small study effects, the overall quality of the CRC body of evidence per the GRADE approach is low.

#### 3.1.2. Leukemia

**Figure 3:**
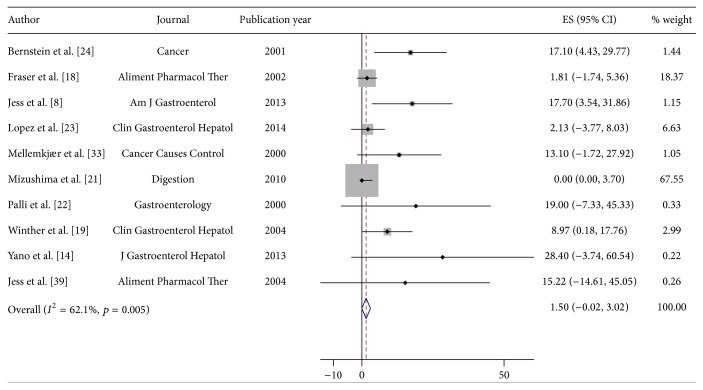
Incidence of leukemia in patients with inflammatory bowel disease (IBD). Each incidence estimate is presented followed by the 95% confidence intervals (CIs). Each square in the plot indicates the point estimate of the incidence. The diamond represents the summary incidence from the pooled studies. Error bars depict the 95% CIs.

Reported incidence rates of leukemia in IBD ranged from 0.0/100,000 py (95% CI 0.0–3.7/100,000) to 28.4/100,000 py (95% CI −3.7–60.5/100,000) (Table 2). Using a random effects model, the pooled estimated incidence of leukemia in IBD of 1.5/100,000 py was obtained (95% CI −0.02–3.0/100,000).  Figure 3 illustrates the Forest plot for the pooled estimates. Moderate between-study heterogeneity was seen (heterogeneity test chi^2^ = 23.8, *p* = 0.005; *I* ^2^ = 62.1%); however this is likely influenced by the small number of available studies. In CD, the range of reported incidence rates was identical to that of IBD (Table 2). In UC, reported incidence rates ranged from 8.97/100,000 py (95% CI 0.2–17.8/100,000) to 25.4/100,000 py (95% CI −9.8–60.6/100,000) (Table 2). The pooled incidence estimate was 0.3/100,000 py for CD (95% CI −1.0–1.6/100,000) and 13.0/100,000 py for UC (95% CI 5.8–20.3/100,000). The *I* ^2^ statistics are 44.3% (heterogeneity test, chi^2^ = 10.8, *p* = 0.096) and 0.0% (heterogeneity test, chi^2^ = 2.65, *p* = 0.449), respectively, indicating low levels of heterogeneity; however the power of this analysis is severely limited due to the small number of included studies.

Stratification by publication year and region did not impact the incidence estimates for IBD or for CD and UC separately. Furthermore, no significant effects of any study- or subject-level covariates on incidence estimates were discovered in metaregression analyses; however the small sample size again restricted the power of these tests.

As less than 10 studies were included, the interpretation of funnel plot symmetry and Egger's test to assess the presence of small study effects and/or publication bias are not recommended [52]. The overall quality of the leukemia body of evidence, per the GRADE approach, is low due to study designs and small sample size.

#### 3.1.3. Lymphoma

Reported incidence rates for lymphoma in IBD ranged from 0.0/100,000 py (95% CI 0.0–3.7/100,000) to 81.7/100,000 py (95% CI 21.2–142.2/100,000) (Table 3). Substantial heterogeneity between studies prevented pooling of estimates (heterogeneity test, chi^2^ = 591.1; *p* < 0.001; *I* ^2^ = 96.6%). Thus, the included studies are presented as unpooled estimates. A sensitivity analysis excluding the two studies with the lowest individual incidence estimates and highest weights on the pooled estimates was conducted, with no significant corresponding decrease in heterogeneity [14, 21].

**Figure 4:**
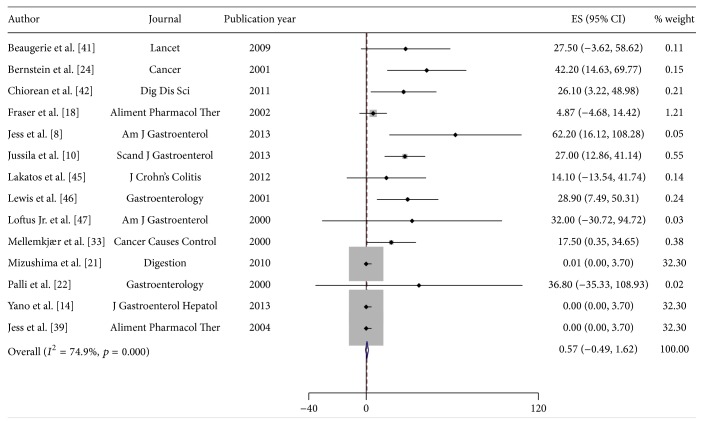
Incidence of lymphoma in patients with Crohn's Disease (CD). Each incidence estimate is presented followed by the 95% confidence intervals (CIs). Each square in the plot indicates the point estimate of the incidence. The diamond represents the summary incidence from the pooled studies. Error bars depict the 95% CIs.

Reported incidence rates of lymphoma in CD ranged from 0.0/100,000 py (95% CI 0.0–3.7/100,000) to 62.2/100,000 py (95% CI 16.1–108.3/100,000) (Table 3). For UC, the incidence rates ranged from 0.0/100,000 py (95% CI 0.0–3.7/100,000) to 76.2/100,000 py (95% CI 15.2–137.2/100,000) (Table 3). A pooled incidence rate of 0.6/100,000 py (95% CI −0.5–1.6/100,000) for CD was obtained. Substantial heterogeneity prevented pooling of estimates for UC (heterogeneity test, chi^2^ = 199.5; *p* < 0.001; *I* ^2^ = 94.5%). A sensitivity analysis excluding the study with the largest impact on the pooled estimate in UC [47] decreased the heterogeneity (heterogeneity test, chi^2^ = 44.79; *p* < 0.001; *I* ^2^ = 77.7%). However, substantial heterogeneity remained, and results for UC are presented as unpooled estimates (Figure 4).

Incidence estimates stratified by publication year and region did not differ. Metaregression analysis revealed a statistically significant effect of age on lymphoma incidence in IBD. For each mean year increase in age, the incidence of lymphoma increased by approximately 2.1/100,000 py (95% CI 0.74–3.4/100,000), explaining approximately 65.8% of the between-study heterogeneity (adjusted *R* ^2^ = 65.8%). No other covariate effects were found in metaregression analyses.

There was weak evidence of publication bias and/or small study effects in the IBD analysis (*p* = 0.213) and in the UC analysis (*p* = 0.824). The number of included studies for CD is less than 10; thus analyses of funnel plots and Egger's test are not recommended [52]. The overall quality of the lymphoma body of evidence, per the GRADE approach, is low due to the observational designs of available studies.

### 3.2. Discussion

This meta-analysis was performed in order to produce updated and reliable incidence rates for CRC, leukemia, and lymphoma in IBD patients and in CD and UC separately. We aimed to quantify cancer incidence associated with underlying IBD, without the effects of immunomodulator and biologic pharmacotherapy, but this was difficult without reliable reporting of treatment information in the available studies. Although we could not pool estimates of the incidence of CRC in IBD and UC specifically, a pooled incidence rate of 53.3/100,000 py (95% CI 46.3–60.3/100,000) in CD was obtained. The estimated worldwide CRC incidence rate is 19.3/100,000 py [53]. In more developed regions of the world, which compares to the regions of origin of the included studies, the incidence rate is higher at 59.2/100,000 py [53]. As such, CRC incidence in CD does not appear to be higher than that of the general population in similar areas of origin. Of note, these incidence estimates are crude (not age-adjusted) and therefore may not reflect differences in the age of the underlying populations.

For leukemia, pooled incidence rates of 1.5/100,000 py (95% CI −0.06–3.0/100,000), 0.3/100,000 py (95% CI −1.0–1.6/100,000), and 13.0/100,000 py (95% CI 5.8–20.3/100,000) were obtained for IBD, CD, and UC, respectively. The estimated worldwide leukemia incidence is 5.0/100,000 py and 11.3/100,000 py in developed regions [53]. Thus, the incidence of leukemia in IBD and CD is lower than that of the general population in developed regions but is slightly higher in UC. For lymphoma, substantial heterogeneity prevented the pooling of estimates for IBD and UC; however a pooled incidence rate of 0.6/100,000 py (95% CI −0.4–2.1/100,000) in CD was obtained. Estimated worldwide lymphoma incidence is 6.4/100,000 py and 17.6/100,000 py in more developed areas [53]. Thus, the incidence of lymphoma in CD is lower than estimated both worldwide and in developed regions.

Due to incomplete reporting of use of immunomodulators and biologics in the published literature, we could not calculate incidence rates of CRC, leukemia, and lymphoma specifically in persons not treated with these medications; however incidence estimates stratified by publication year before and after widespread use of these medications were not significantly different. This suggests that the impact of immunomodulators and biologics on the incidence of these cancers may be negligible. Metaregression did not reveal any significant subject- or study-level covariate effects in the majority of analyses, with the exception of the effect of mean age on the incidence of lymphoma in IBD. The power of these tests was limited by incomplete reporting of these variables and the small number of included studies.

The strength of the present study is the comprehensiveness of the literature search and evaluation of data for inclusion. Despite the exhaustiveness of the search, we could include only a small number of studies, limiting the power of the pooled analyses and ultimate confidence in incidence estimates. In addition, substantial heterogeneity prevented pooling of estimates in some cases. The heterogeneity of the included studies may reflect differences in follow-up time, cohort size, geographic differences in patient care, or other factors that we were unable to assess due to incomplete reporting in the published literature. Although these limitations may lead to bias in our incidence estimates, the direction of which is indeterminable, our estimates are based on the best available evidence.

## 4. Conclusions

This meta-analysis presents updated estimates of the incidence of CRC, leukemia, and lymphoma in adults with IBD. Overall, the incidence of these malignancies does not appear to be higher than in the general population. Further research is needed to explore patient characteristics that may modify the risk of malignancy. Specifically, we need large population based cohort studies in IBD patients that report complete demographic and outcome data. Detailed information on immunomodulator and biologic use is limited in the published literature, and if we are to be able to truly understand the potential increased risk of malignancy associated with IBD pharmacotherapy, this information is required.

## Supplementary Material

Supplementary Table 1 details the search algorithms used for the systematic review. The search algorithm specific to each source are listed along with the number of results returned.

## Figures and Tables

**(a):** 

Author	Journal	Publication year	Study design	Study population	Region of origin	Number of sites	Study duration (yrs)	Person-years	Number of patients	Diagnosis	Mean age (yrs)	Female (%)	Mean disease duration (yrs)	Surgery (%)
Askling et al. [25]	Gastroenterology	2001	Cohort	National registry	Europe (Western)	Countrywide	54	169,332 *91,833* *77,499 *	19,459 *8,810* *10,649 *	IBD *CD* *UC*		48.6 *53.0* *45.0*		

Bernstein et al. [24]	Cancer	2001	Case-control	Administrative claims	Canada	Regionwide	14	41,005 *21,340* *19,665 *	5,529 *2,857* *2,672 *	IBD *CD* *UC*	39.0 *36.3* *41.7*	54.5 *59.0* *50.0*		

Farrell et al. [26]	Gut	2000	Cohort	Referral center	Europe (Western)	1	9	6,256	782	IBD^*∗*^	44.1	52.0	10.0	

Fraser et al. [18]	Aliment Pharmacol Ther	2002	Cohort	Referral center	Europe (Western)	Countywide	35	55,388 *20,494* *34,894 *	1,578 *584* *994 *	IBD *CD* *UC*	35.0	53.0		

Gillen et al. [27]	Gut	1994	Cohort	Referral center	Europe (Western)	Countrywide	30	12,324 *2,320* *10,004 *	611 *125* *486 *	IBD *CD* *UC*				

Herrinton et al. [28]	Gastroenterology	2012	Cohort	Administrative claims	United States	Countywide	12	61,793 *28,469* *33,324 *	14,875 *5,053* *9,822 *	IBD *CD* *UC*	61.8 *62.4* *61.1*			

Hou et al. [29]	Inflamm Bowel Dis	2012	Cohort	National registry	United States	Countrywide	11	112,243	20,949	UC	61.6	5.0	5.0	

Jess et al. [7]	Gastroenterology	2012	Cohort	National registry	Europe (Western)	Countywide	29	385,608 *130,391* *255,217 *	47,374 *14,463* *32,911 *	IBD *CD* *UC*	40.3 *35.7* *44.9*	55.0 *57.0* *53.0*		

Jess et al. [39]	Aliment Pharmacol Ther	2004	Cohort	Regional registry	Europe (Western)	Regionwide	35	6,569	374	CD		58.0		

Jussila et al. [10]	Scand J Gastroenterol	2013	Cohort	National registry	Europe (Western)	Countrywide	23	232,536 *51,876* *180,660 *	20,970 *4,983* *15,987 *	IBD *CD* *UC*				

Lakatos et al. [38]	Inflamm Bowel Dis	2006	Cohort	Provincial registry	Europe (Eastern)	7	11	8,564	723	UC	49.0	47.0	10.0	

Lakatos et al. [30]	J Crohn's Colitis	2011	Cohort	Provincial registry	Europe (Eastern)	7	31	5,758	506	CD	31.5	50.4		31.0

Lennard-Jones et al. [31]	Gut	1990	Cohort	Surveillance	Europe (Western)	1	21	4,048	401	UC		42.6		

Lovasz et al. [32]	J Gastroenterol Liver Dis	2013	Cohort	Provincial registry	Europe (Eastern)	Regionwide	34	7,759	640	CD	28.0	49.8	11.0	38.4

Manninen et al. [11]	J Crohn's Colitis	2013	Cohort	Referral center	Europe (Western)	1	21	22,900 *7,265* *15,635 *	1,804 *551* *1,253 *	IBD *CD* *UC*	33.0 *30.0* *34.0*	47.0 *51.0* *45.0*	13.5 *13.0* *13.1*	46.0

Mellemkjær et al. [33]	Cancer Causes Control	2000	Cohort	National registry	Europe (Western)	Countrywide	16	22,875	2,645	CD		50.0		

Mizushima et al. [21]	Digestion	2010	Cohort	Referral center	Asia	1	20	4,248	294	CD	39.0	30.6		

Palli et al. [22]	Gastroenterology	2000	Cohort	Regional registry	Europe (Western)	1	19	10,592 *2,716* *7,877 *	920 *231* *689 *	IBD *CD* *UC*				

Pasternak et al. [34]	Am J Epidemiology	2013	Cohort	National registry	Europe (Western)	Countrywide	11	304,992	38,772	IBD^*∗*^	47.0	55.0		4.0

Selinger et al. [13]	Clin Gastroenterol Hepatol	2014	Cohort	Referral center	Australia/New Zealand	2	15	13,423 *5,417* *8,006 *	881 *377* *504 *	IBD *CD* *UC*	31.5 *29.0* *34.0*	53.1 *59.1* *47.1*		

van Schaik et al. [35]	Gut	2012	Cohort	National registry	Europe (Western)	Countrywide	8	4,864	835	IBD^*∗*^	43.0	57.0	2.9	

Venkataraman et al. [36]	Australian J Gastroenterol Hepatol	2005	Cohort	Referral center	Asia	1	25	4,901	532	UC		36.8	6.0	8.8

Wandall et al. [37]	Scand J Gastroenterol	2000	Cohort	Regional registry	Europe (Western)	Regionwide	25	8,101	801	UC	41.0	44.8	10.1	15.9

Winther et al. [19]	Clin Gastroenterol Hepatol	2004	Cohort	Regional registry	Europe (Western)	Regionwide	35	22,290	1,160	UC		53.4		

Yano et al. [14]	J Gastroenterol Hepatol	2013	Cohort	Referral center	Asia	1	25	10,552	770	CD	25.1	31.3	13.1	

^*∗*^Did not report separate incidence estimates for CD and UC.

**(b):** 

Author	Journal	Publication year	PSC (%)	Pancolitis (%)	Immunomodulator use (%)	Biologic use (%)	Observed number of CRCs	Incidence rate (per 100,000 persons)	Standard error	95% CI lower bound	95% CI upper bound	Bias rating
Askling et al. [25]	Gastroenterology	2001		10.3 *17.0* *4.8*			143 *40* *103*	84.4 *43.6* *132.9*	7.1 *6.9* *13.1*	70.6 *30.1* *107.2*	98.2 *57.1* *158.6*	Moderate

Bernstein et al. [24]	Cancer	2001			0.0 *0.0* *0.0*	0.0 *0.0* *0.0*	60 *24* *36*	146.3 *112.5* *183.1*	18.9 *23.0* *30.5*	109.3 *67.5* *123.3*	183.3 *157.5* *242.9*	Moderate

Farrell et al. [26]	Gut	2000		26.0	30.0		3	48.0	27.7	−6.3	102.3	Moderate

Fraser et al. [18]	Aliment Pharmacol Ther	2002		30.0	0.0 *0.0* *0.0*	0.0 *0.0* *0.0*	23 *4* *19*	41.5 *19.5* *54.5*	8.7 *9.8* *12.5*	24.5 *0.4* *30.0*	58.5 *38.6* *79.0*	Moderate

Gillen et al. [27]	Gut	1994					37 *8* *29*	300.2 *344.9* *289.9*	49.4 *121.9* *53.8*	203.5 *105.9* *184.4*	396.9 *583.9* *395.4*	Moderate

Herrinton et al. [28]	Gastroenterology	2012					82 *29* *53*	132.7 *101.9* *159.0*	14.7 *18.9* *21.8*	104.0 *64.8* *116.2*	161.4 *139.0* *201.8*	Moderate

Hou et al. [29]	Inflamm Bowel Dis	2012					183	163.0	12.0	139.4	186.6	Moderate

Jess et al. [7]	Gastroenterology	2012					338 *70* *268*	87.7 *53.7* *105.0*	4.8 *6.4* *6.4*	78.4 *41.1* *92.4*	97.0 *66.3* *117.6*	Moderate

Jess et al. [39]	Aliment Pharmacol Ther	2004					4	60.9	30.4	1.2	120.6	Moderate

Jussila et al. [10]	Scand J Gastroenterol	2013					189 *32* *157*	81.3 *61.7* *86.9*	5.9 *10.9* *6.9*	69.7 *40.3* *73.3*	92.9 *83.1* *100.5*	Moderate

Lakatos et al. [38]	Inflamm Bowel Dis	2006	2.9	25.8			13	151.8	42.1	69.3	234.3	Moderate

Lakatos et al. [30]	J Crohn's Colitis	2011	1.8				5	86.8	38.8	10.7	162.9	Moderate

Lennard-Jones et al. [31]	Gut	1990					22	543.5	115.9	316.4	770.6	Moderate

Lovasz et al. [32]	J Gastroenterol Liver Dis	2013	0.9	34.5	47.2	7.7	6	77.3	31.6	15.4	139.2	Moderate

Manninen et al. [11]	J Crohn's Colitis	2013	2.5 *1.1* *3.2*	43.2 *37.7* *49.4*			21 *5* *16*	91.7 *68.8* *102.3*	20.0 *30.8* *25.6*	52.5 *8.5* *52.2*	130.9 *129.1* *152.4*	Moderate

Mellemkjær et al. [33]	Cancer Causes Control	2000					15	65.6	16.9	32.4	98.8	Moderate

Mizushima et al. [21]	Digestion	2010		12.4			6	141.2	57.6	28.2	254.2	Moderate

Palli et al. [22]	Gastroenterology	2000					12 *2* *10*	113.0 *73.6* *127.0*	32.6 *52.0* *40.2*	49.1 *−28.4* *48.3*	176.9 *175.6* *205.7*	Moderate

Pasternak et al. [34]	Am J Epidemiology	2013			0.0	0.0	380	124.6	6.4	112.1	137.1	Moderate

Selinger et al. [13]	Clin Gastroenterol Hepatol	2014		38.4 *37.4* *39.1*			29 *5* *24*	216.0 *92.3* *299.8*	40.1 *41.3* *61.2*	137.4 *11.4* *179.9*	294.6 *173.2* *419.7*	Moderate

van Schaik et al. [35]	Gut	2012		29.0	0.0	0.0	9	185.0	61.7	64.1	305.9	Moderate

Venkataraman et al. [36]	Australian J Gastroenterol Hepatol	2005		44.0			5	102.0	45.6	12.6	191.4	Moderate

Wandall et al. [37]	Scand J Gastroenterol	2000		18.0			6	74.1	30.3	14.8	133.4	Moderate

Winther et al. [19]	Clin Gastroenterol Hepatol	2004		54.0			13	58.3	16.2	26.6	90.0	Moderate

Yano et al. [14]	J Gastroenterol Hepatol	2013		14.7			9	85.3	28.4	29.6	141.0	Moderate

**(a):** 

Author	Journal	Publication year	Study design	Study population	Region of origin	Number of sites	Study duration (yrs)	Person-years	Number of patients	Diagnosis	Mean age (yrs)	Female (%)	Mean disease duration (yrs)	Surgery (%)
Bernstein et al. [24]	Cancer	2001	Case-control	Administrative claims	Canada	Regionwide	14	41,005 *21,340* *19,665*	5,529 *2,857* *2,672*	IBD *CD* *UC*	39.0 *36.3* *41.7*	54.5 *59.0* *50.0*		

Fraser et al. [18]	Aliment Pharmacol Ther	2002	Cohort	Referral center	Europe (Western)	Countrywide	35	55,388	1,578	IBD^*∗*^	35.0	53.0		

Jess et al. [8]	Am J Gastroenterol	2013	Cohort	Regional registry	Europe (Western)	1	32	33,843 *11,261* *22,582*	2,211 *774* *1,437*	IBD *CD* *UC*		53.0 *57.0* *49.0*		

Jess et al. [39]	Aliment Pharmacol Ther	2004	Cohort	Regional registry	Europe (Western)	Regionwide	35	6,569	374	CD		58.0		

Lopez et al. [23]	Clin Gastroenterol Hepatol	2014	Cohort	National registry	Europe (Western)	Countrywide	3	23,457	10,810	IBD^*∗*^	40.0	53.0		

Mellemkjær et al. [33]	Cancer Causes Control	2000	Cohort	National registry	Europe (Western)	Countrywide	16	22,875	2,645	CD		50.0		

Mizushima et al. [21]	Digestion	2010	Cohort	Referral center	Asia	1	20	4,248	294	CD	39.0	30.6		

Palli et al. [22]	Gastroenterology	2000	Cohort	Regional registry	Europe (Western)	1	19	10,592 *2,716* *7,877*	920 *231* *689*	IBD *CD* *UC*				

Winther et al. [19]	Clin Gastroenterol Hepatol	2004	Cohort	Regional registry	Europe (Western)	Regionwide	35	22,290	1,160	UC		53.4		

Yano et al. [14]	J Gastroenterol Hepatol	2013	Cohort	Referral center	Asia	1	25	10,552	770	CD	25.1	31.3	13.1	

**(b):** 

Author	Journal	Publication year	PSC (%)	Pancolitis (%)	Immunomodulator use (%)	Biologic use (%)	Observed number of leukemia cases	Incidence rate (per 100,000 persons)	Standard error	95% CI lower bound	95% CI upper bound	Bias rating
Bernstein et al. [24]	Cancer	2001			0.0 *0.0* *0.0*	0.0 *0.0* *0.0*	7 *3* *4*	17.1 *14.1* *20.3*	6.5 *8.1* *10.2*	4.4 *−1.9* *0.4*	29.8 *30.1* *40.2*	Moderate

Fraser et al. [18]	Aliment Pharmacol Ther	2002		30.0	0.0	0.0	1	1.81	1.8	−1.7	5.4	Moderate

Jess et al. [8]	Am J Gastroenterol	2013		26.7 *41.0* *19.0*	27.2 *45.0* *18.0*		6 *1* *5*	17.7 *8.9* *22.1*	7.2 *8.9* *9.9*	3.5 *−8.5* *2.7*	31.9 *26.3* *41.5*	Moderate

Jess et al. [39]	Aliment Pharmacol Ther	2004					1	15.2	15.2	−14.6	45.1	Moderate

Lopez et al. [23]	Clin Gastroenterol Hepatol	2014			0.0	0.0	0.5	2.13	3.0	−3.8	8.0	Moderate

Mellemkjær et al. [33]	Cancer Causes Control	2000					3	13.1	7.6	−1.7	27.9	Moderate

Mizushima et al. [21]	Digestion	2010		12.4			0	0.0		0.0	3.7	Moderate

Palli et al. [22]	Gastroenterology	2000					2 *0* *2*	19.0 *0.0* *25.4*	13.4 *18.0*	−7.3 *0.0* *−9.8*	45.3 *3.7* *60.6*	Moderate

Winther et al. [19]	Clin Gastroenterol Hepatol	2004		54.0			4	8.97	4.5	0.2	17.8	Moderate

Yano et al. [14]	J Gastroenterol Hepatol	2013		14.7			3	28.4	16.4	−3.7	60.5	Moderate

^*∗*^Did not report separate incidence measures for CD and UC.

**(a):** 

Author	Journal	Publication year	Study design	Study population	Region of origin	Number of sites	Study duration (yrs)	Person-years	Number of patients	Diagnosis	Mean age (yrs)	Female (%)	Mean disease duration (yrs)	Surgery (%)
Abbas et al. [40]	Am J Gastroenterol	2012	Cohort	National registry	United States	Countrywide	11.0	352,429	32,039	UC	60.0	7.0		

Beaugerie et al. [41]	Lancet	2009	Cohort	National registry	Europe (Western)	Countrywide	3.0	22,706 * 10,899 * * 11,807 *	10,810 * 5,153 * * 5,657 *	IBD *CD* *UC*		53.0		

Bernstein et al. [24]	Cancer	2001	Case-control	Administrative claims	Canada	Regionwide	14.0	41,005 * 21,340 * * 19,665 *	5,529 * 2,857 * * 2,672 *	IBD *CD* *UC*	39.0 *36.3* *41.7*	54.5 *59.0* *50.0*		

Chiorean et al. [42]	Dig Dis Sci	2011	Case-control	Referral center	United States	1	8.4	30,121 * 19,127 * * 10,994 *	3,585 * 2,277 * * 1,308 *	IBD *CD* *UC*				

Farrell et al. [26]	Gut	2000	Cohort	Referral center	Europe (Western)	1	9.0	6,256	782	IBD^*∗*^	44.1	52.0	10.0	

Fraser et al. [18]	Aliment Pharmacol Ther	2002	Cohort	Referral center	Europe (Western)	Countrywide	35.0	55,388 * 20,494 * * 34,894 *	1,578 * 584 * * 994 *	IBD *CD* *UC*	35.0	53.0		

Herrinton et al. [43]	Am J Gastroenterol	2011	Cohort	Administrative claims	United States	Regionwide	13.0	67,867	16,023	IBD^*∗*^		53.0		

Jess et al. [8]	Am J Gastroenterol	2013	Cohort	Regional registry	Europe (Western)	1	32.0	33,843 * 11,261 * * 22,582 *	2,211 * 774 * * 1,437 *	IBD *CD* *UC*		53.0 *57.0* *49.0*		

Jess et al. [39]	Aliment Pharmacol Ther	2004	Cohort	Regional registry	Europe (Western)	Regionwide	35.0	6,569	374	CD		58.0		

Jussila et al. [10]	Scand J Gastroenterol	2013	Cohort	National registry	Europe (Western)	Countrywide	23.0	232,536 *51,876 * *180,660 *	20,970 * 4,983 * * 15,987 *	IBD *CD* *UC*				

Khan et al. [44]	Gastroenterology	2013	Cohort	National registry	United States	Countrywide	10.0	199,046	36,891	UC	60.0	7.0		

Lakatos et al. [45]	J Crohn's Colitis	2012	Cohort	Provincial registry	Europe (Eastern)	7	31.0	19,293 * 7,093 * * 12,830 *	1,420 * 506 * * 914 *	IBD *CD* *UC*	32.5 *28.5* *36.5*	48.8 *50.0* *47.6*		22.8 *41.3* *4.2*

Lewis et al. [46]	Gastroenterology	2001	Cohort	National registry	Europe (Western)	Countrywide	9.0	64,239 * 24,221 * * 40,018 *	16,996 * 6,605 * * 10,391 *	IBD *CD* *UC*	47.3 *44.3* *50.3*	54.0 *58.0* *50.0*		

Loftus Jr. et al. [47]	Am J Gastroenterol	2000	Cohort	Regional registry	United States	2	53.0	6,662 * 3,150 * * 3,512 *	454 * 216 * * 238 *	IBD *CD* *UC*		24.0	14.9	

Mellemkjær et al. [33]	Cancer Causes Control	2000	Cohort	National registry	Europe (Western)	Countrywide	16.0	22,875	2,645	CD		50.0		

Mizushima et al. [21]	Digestion	2010	Cohort	Referral center	Asia	1	20.0	4,248	294	CD	39.0	30.6		

Palli et al. [22]	Gastroenterology	2000	Cohort	Regional registry	Europe (Western)	1	19.0	10,592 * 2,716 * * 7,877 *	920 * 231 * * 689 *	IBD *CD* *UC*				

Pasternak et al. [34]	Am J Epidemiology	2013	Cohort	National registry	Europe (Western)	Countrywide	11.0	304,992	38,772	IBD^*∗*^	47.0	55.0		4.0

Van Domselaar et al. [48]	J Gastroenterol Hepatol	2010	Cohort	Referral center	Europe (Western)	1		8,563	911	IBD^*∗*^	53.0	28.6	4.8	

Winther et al. [19]	Clin Gastroenterol Hepatol	2004	Cohort	Regional registry	Europe (Western)	Regionwide	35.0	22,290	1,160	UC		53.4		

Yano et al. [14]	J Gastroenterol Hepatol	2013	Cohort	Referral center	Asia	1	25.0	10,552	770	CD	25.1	31.3	13.1	

**(b):** 

Author	Journal	Publication year	PSC (%)	Pancolitis (%)	Immunomodulator use (%)	Biologic use (%)	Observed number of lymphomas	Incidence rate (per 100,000 persons)	Standard error	95% CI lower bound	95% CI upper bound	Bias rating
Abbas et al. [40]	Am J Gastroenterol	2012			0.0	0.0	282	80.0	4.8	70.7	89.3	Moderate

Beaugerie et al. [41]	Lancet	2009		29.6 *13.0* *16.0*	0.0 *0.0* *0.0*	0.0 *0.0* *0.0*	6 *3* *3*	26.4 *27.5* *25.4*	10.8 *15.9* *14.7*	5.3 *−3.6* *−3.3*	47.5 *58.6* *54.1*	Moderate

Bernstein et al. [24]	Cancer	2001			0.0 *0.0* *0.0*	0.0 *0.0* *0.0*	16 *9* *7*	39.0 *42.2* *35.6*	9.8 *14.1* *13.5*	19.9 *14.6* *9.2*	58.1 *69.8* *62.0*	Moderate

Chiorean et al. [42]	Dig Dis Sci	2011					8 *5* *3*	26.6 *26.1* *27.3*	9.4 *11.7* *15.8*	8.2 *3.2* *−3.6*	45.0 *49.0* *58.2*	Moderate

Farrell et al. [26]	Gut	2000		26.0	30.0		4	64.0	32.0	1.3	126.7	Moderate

Fraser et al. [18]	Aliment Pharmacol Ther	2002		30.0	0.0 *0.0* *0.0*	0.0 *0.0* *0.0*	5 *1* *4*	9.0 *4.87* *11.5*	4.0 *4.9* *5.8*	1.1 *−4.7* *0.2*	16.9 *14.4* *22.8*	Moderate

Herrinton et al. [43]	Am J Gastroenterol	2011			0.0	0.0	33	48.6	8.5	32.0	65.2	Moderate

Jess et al. [8]	Am J Gastroenterol	2013		26.7 *41.0* *19.0*	27.2 *45.0* *18.0*		15 *7* *8*	44.3 *62.2* *35.4*	11.4 *23.5* *12.5*	21.9 *16.1* *10.9*	66.7 *108.3* *59.9*	Moderate

Jess et al. [39]	Aliment Pharmacol Ther	2004					0	0.0		0.0	3.7	Moderate

Jussila et al. [10]	Scand J Gastroenterol	2013					72 *14* *58*	31.0 *27.0* *32.1*	3.7 *7.2* *4.2*	23.8 *12.9* *23.8*	38.2 *41.1* *40.4*	Moderate

Khan et al. [44]	Gastroenterology	2013			0.0	0.0	119	60.0	5.5	49.2	70.8	Moderate

Lakatos et al. [45]	J Crohn's Colitis	2012	2.3 *1.8* *2.7*	30.2 *35.9* *24.4*	0.0 *0.0* *0.0*	0.0 *0.0* *0.0*	3 *1* *2*	15.5 *14.1* *15.6*	8.9 *14.1* *11.0*	−2.0 *−13.5* *−6.0*	33.0 *41.7* *37.2*	Moderate

Lewis et al. [46]	Gastroenterology	2001			9.5 *13.0* *6.0*		18 *7* *11*	28.0 *28.9* *27.5*	6.6 *10.9* *8.3*	15.1 *7.5* *11.2*	40.9 *50.3* *43.8*	Moderate

Loftus Jr. et al. [47]	Am J Gastroenterol	2000					1 *1* *0*	15.0 *32.0* *0.0*	15.0 *32.0*	−14.4 *−30.7* *0.0*	44.4 *94.7* *3.7*	Moderate

Mellemkjær et al. [33]	Cancer Causes Control	2000					4	17.5	8.8	0.4	34.7	Moderate

Mizushima et al. [21]	Digestion	2010		12.4			0	0.0		0.0	3.7	Moderate

Palli et al. [22]	Gastroenterology	2000					7 *1* *6*	66.0 *36.8* *76.2*	24.9 *36.8* *31.1*	17.1 *−35.3* *15.2*	114.9 *108.9* *137.2*	Moderate

Pasternak et al. [34]	Am J Epidemiology	2013			0.0	0.0	46	15.1	2.2	10.7	19.5	Moderate

Van Domselaar et al. [48]	J Gastroenterol Hepatol	2010					7	81.7	30.9	21.2	142.2	Moderate

Winther et al. [19]	Clin Gastroenterol Hepatol	2004		54.0			2	17.9	12.7	−6.9	42.8	Moderate

Yano et al. [14]	J Gastroenterol Hepatol	2013		14.7			0	0.0		0.0	3.7	Moderate

^*∗*^Did not report separate incidence estimates for CD and UC.

## Abbreviations

CRC: Colorectal cancer
IBD: Inflammatory bowel disease
PSC: Primary sclerosing cholangitis
LD: Lymphoproliferative disorders
CD: Crohn's Disease
UC: Ulcerative colitis
CI: Confidence interval
py: Person-years.
