# Supplementary material for: Worldwide Incidence of Colorectal Cancer, Leukemia, and Lymphoma in Inflammatory Bowel Disease: An Updated Systematic Review and Meta-Analysis
Source: Gastroenterol Res Pract. 2016 May 16;2016:1632439. doi: 10.1155/2016/1632439 (PMC4884856; doi:10.1155/2016/1632439)
Supplement: Supplementary file 1 — Supplementary Table 1 details the search algorithms used for the systematic review. The search algorithm specific to each source are listed along with the number of results returned. [file 1632439.f1.docx]

**Supplementary Table 1**

Supplementary Table 1-Search Algorithms

| **PUBMED (results returned=2655)** |
| --- |
| ((((((((((((inflammatory bowel diseases[MeSH Terms]) OR inflammatory bowel disease[Title]) OR inflammatory bowel diseases[Title]) OR “crohn's disease”[Title]) OR “crohns disease”[Title]) OR “crohn disease”[Title]) OR “ulcerative colitis”[Title]) OR colitis[Title]) OR enterocolitis[Title]) OR ileocolitis[Title]) OR ileitis[Title])) AND (((((((((((((((((((Digestive System Neoplasms[MeSH Terms]) OR Abdominal Neoplasms[MeSH Terms]) OR Lymphoma[MeSH Terms]) OR Leukemia[MeSH Terms]) OR “colorectal cancer”[Title/Abstract]) OR “colorectal carcinoma”[Title/Abstract]) OR “colorectal tumors”[Title/Abstract]) OR “colorectal neoplasms” [Title/Abstract]) OR “colonic neoplasms”[Title/Abstract]) OR “rectal neoplasms”[Title/Abstract]) OR “rectal cancer”[Title/Abstract]) OR “colon cancer”[Title/Abstract]) OR lymphoma[Title/Abstract]) OR “hodgkin disease”[Title/Abstract]) OR “nonhodgkin lymphoma”[Title/Abstract]) OR lymphosarcoma[Title/Abstract]) OR leukemia[Title/Abstract]) OR leucocythaemia[Title/Abstract]) OR leucocythemia[Title/Abstract])))) AND adult) AND human |
| **EMBASE (results returned=574)** |
| ('inflammatory bowel diseases':ti OR 'crohns disease':ti OR 'ulcerative colitis':ti OR colitis:ti OR enterocolitis:ti OR ileitis:ti OR ileocolitis:ti) AND ('digestive system neoplasms':ab,ti OR 'abdominal neoplasms':ab,ti OR lymphoma:ab,ti OR leukemia:ab,ti OR 'colorectal cancer':ab,ti OR 'colorectal carcinoma':ab,ti OR 'colorectal tumors':ab,ti OR 'colonic neoplasms':ab,ti OR 'rectal neoplasms':ab,ti OR 'rectal cancer':ab,ti OR 'colon cancer':ab,ti OR 'hodgkin disease':ab,ti OR 'nonhodgkin lymphoma':ab,ti OR lymphosarcoma:ab,ti OR leucocythaemia:ab,ti OR leucocythemia:ab,ti) AND (1990:py OR 1991:py OR 1992:py OR 1993:py OR 1994:py OR 1995:py OR 1996:py OR 1997:py OR 1998:py OR 1999:py OR 2000:py OR 2001:py OR 2002:py OR 2003:py OR 2004:py OR 2005:py OR 2006:py OR 2007:py OR 2008:py OR 2009:py OR 2010:py OR 2011:py OR 2012:py OR 2013:py OR 2014:py) AND ('adult') AND ('human') |
| **SCOPUS (results returned=18)** |
| ( TITLE ( inflammatory bowel disease* OR crohn* disease OR ulcerative colitis OR colitis OR enterocolitis OR ileitis OR ileocolitis ) AND TITLE-ABS-KEY ( digestive system neoplasm* ) OR TITLE-ABS-KEY ( abdominal neoplasm* ) OR TITLE-ABS-KEY ( lymphoma ) OR TITLE-ABS-KEY ( leukemia ) OR TITLE-ABS-KEY ( colorectal cancer ) OR TITLE-ABS-KEY ( colorectal carcinoma ) OR TITLE-ABS-KEY ( colorectal tumor* ) OR TITLE-ABS-KEY ( colorectal neoplasm* ) OR TITLE-ABS-KEY ( colonic neoplasm* ) OR TITLE-ABS-KEY ( rectal neoplasm* ) OR TITLE-ABS-KEY ( rectal cancer ) OR TITLE-ABS-KEY ( colon cancer ) OR TITLE-ABS-KEY ( hodgkin disease ) OR TITLE-ABS-KEY ( nonhodgkin lymphoma ) OR TITLE-ABS-KEY ( lymphosarcoma ) OR TITLE-ABS-KEY ( leucocythaemia ) OR TITLE-ABS-KEY ( leucocythemia ) ) AND PUBYEAR > 1989 AND  TITLE-ABS-KEY ( adult )  AND  TITLE-ABS-KEY ( human ) |
| **COCHRANE LIBRARY (results returned=33)** |
| "inflammatory bowel disease" OR "crohn’s disease" OR "ulcerative colitis" OR colitis OR enterocolitis OR ileitis OR ileocolitis in Record Title and "digestive system neoplasm" OR "abdominal neoplasm" OR lymphoma OR leukemia OR "colorectal cancer" OR "colorectal carcinoma" OR "colorectal tumor" OR "colorectal neoplasm" OR "colonic neoplasm" OR "rectal neoplasm" OR "rectal cancer" OR "colon cancer" OR "hodgkin disease" OR "nonhodgkin lymphoma" OR lymphosarcoma OR leucocythaemia OR leucocythemia in Title, Abstract, Keywords and "adult" |
| **WEB OF SCIENCE (results returned=120)** |
| TI=(inflammatory bowel disease* OR crohns disease OR ulcerative colitis OR colitis OR enterocolitis OR ileitis OR ileocolitis) AND TS=(digestive system neoplasm* OR abdominal neoplasm* OR lymphoma OR leukemia OR colorectal cancer OR colorectal carcinoma OR colorectal tumor* OR colorectal neoplasm* OR colonic neoplasm* OR rectal neoplasm* OR rectal cancer OR colon cancer OR hodgkin disease OR nonhodgkin lymphoma OR lymphosarcoma OR leucocythaemia OR leucocythemia) AND TS=(adult) AND TS=(human OR patient OR man OR woman) |
| **MEDLINE (results returned=130)** |
| TI(inflammatory bowel disease* OR crohns disease OR ulcerative colitis OR colitis OR enterocolitis OR ileitis OR ileocolitis) AND TX(digestive system neoplasm* OR abdominal neoplasm* OR lymphoma OR leukemia OR colorectal cancer OR colorectal carcinoma OR colorectal tumor* OR colorectal neoplasm* OR colonic neoplasm* OR rectal neoplasm* OR rectal cancer OR colon cancer OR hodgkin disease OR nonhodgkin lymphoma OR lymphosarcoma OR leucocythaemia OR leucocythemia) AND TX(adult) AND TX(human) |
| **ProQuest Dissertations and Theses (results returned=16)** |
| ti(inflammatory bowel disease* OR crohns disease OR ulcerative colitis OR colitis OR enterocolitis OR ileitis OR ileocolitis) AND all(digestive system neoplasm* OR abdominal neoplasm* OR lymphoma OR leukemia OR colorectal cancer OR colorectal carcinoma OR colorectal tumor* OR colorectal neoplasm* OR colonic neoplasm* OR rectal neoplasm* OR rectal cancer OR colon cancer OR hodgkin disease OR nonhodgkin lymphoma OR lymphosarcoma OR leucocythaemia OR leucocythemia) NOT all((mouse OR rat OR murine)) |
